# Rubisco Evolution in C_4_ Eudicots: An Analysis of Amaranthaceae *Sensu Lato*

**DOI:** 10.1371/journal.pone.0052974

**Published:** 2012-12-20

**Authors:** Maxim V. Kapralov, J. Andrew C. Smith, Dmitry A. Filatov

**Affiliations:** Department of Plant Sciences, University of Oxford, South Parks Road, Oxford, United Kingdom; Montreal Botanical Garden, Canada

## Abstract

**Background:**

Rubisco (ribulose-1,5-bisphosphate carboxylase/oxygenase) catalyses the key reaction in the photosynthetic assimilation of CO_2_. In C_4_ plants CO_2_ is supplied to Rubisco by an auxiliary CO_2_-concentrating pathway that helps to maximize the carboxylase activity of the enzyme while suppressing its oxygenase activity. As a consequence, C_4_ Rubisco exhibits a higher maximum velocity but lower substrate specificity compared with the C_3_ enzyme. Specific amino-acids in Rubisco are associated with C_4_ photosynthesis in monocots, but it is not known whether selection has acted on Rubisco in a similar way in eudicots.

**Methodology/Principal Findings:**

We investigated Rubisco evolution in Amaranthaceae *sensu lato* (including Chenopodiaceae), the third-largest family of C_4_ plants, using phylogeny-based maximum likelihood and Bayesian methods to detect Darwinian selection on the chloroplast *rbcL* gene in a sample of 179 species. Two Rubisco residues, 281 and 309, were found to be under positive selection in C_4_ Amaranthaceae with multiple parallel replacements of alanine by serine at position 281 and methionine by isoleucine at position 309. Remarkably, both amino-acids have been detected in other C_4_ plant groups, such as C_4_ monocots, illustrating a striking parallelism in molecular evolution.

**Conclusions/Significance:**

Our findings illustrate how simple genetic changes can contribute to the evolution of photosynthesis and strengthen the hypothesis that parallel amino-acid replacements are associated with adaptive changes in Rubisco.

## Introduction

Rubisco (ribulose-1,5-bisphosphate carboxylase/oxygenase, EC 4.1.1.39) serves as the main gateway for inorganic carbon to enter metabolic pathways in most ecosystems and hence is unique in its importance to support life. Observations of significant variation in Rubisco kinetics between plant species [1], [2],[3], the correlation of Rubisco kinetics with temperature [4] and CO_2_ availability [5], and positive selection on Rubisco at the molecular level in all principal lineages of land plants [6] support the hypothesis that all Rubiscos may be well adapted to their subcellular environment [7]. However, the molecular mechanisms responsible for optimizing the relationship between Rubisco specificity and its maximum rate of catalytic turnover in particular conditions are still open to debate [8]. Here we use a phylogeny-based approach to investigate how the occurrence of C_4_ photosynthesis has influenced Rubisco evolution at the molecular level in eudicots as represented by the family Amaranthaceae *sensu lato*.

Rubisco discriminates imperfectly between CO_2_ and O_2_ as substrates, and under present-day atmospheric conditions (385 p.p.m. CO_2_), the carboxylase activity of Rubisco is undersaturated in C_3_ plants, and the oxygenase activity gives rise directly to the competing process of photorespiration. Photorespiratory rates in C_3_ plants increase steeply with increasing temperature and give rise to a distinct temperature optimum for net photosynthesis, above which plant yields decline steeply. Increased carbon loss via photorespiration at higher temperatures is attributable mainly to the declining specificity of Rubisco for CO_2_ relative to O_2_ (*S*_c/o_). In fact, it has been proposed that the very slow turnover of Rubisco (*k*_cat_ ≈3 s^−1^) is a direct consequence of the enzyme's particular reaction mechanism, in which *S*_c/o_ is maximized by tight binding of the transition-state intermediate [7]. Land plants also depend on the enzyme rubisco activase which removes tightly binding inhibitors at the active site of Rubisco and thus prevents the loss of its catalytic activity. The cascade of side-reactions performed by Rubisco is yet to be fully understood although recent achievements in mathematical modelling of Rubisco reactions offer the theoretical background for predicting ‘side-effects’ by simulating the overall kinetic behaviour [9]. Another corollary of low *k*_cat_ and of the large size of the holoenzyme (560 kDa) is that Rubisco comprises up to 50% of soluble protein in photosynthetic tissues and is probably the most abundant enzyme on Earth [10].

In terrestrial plants with C_4_ photosynthesis or crassulacean acid metabolism (CAM), and in many aquatic organisms, photorespiration is partially or completely suppressed by the operation of an auxiliary CO_2_-concentrating mechanism. C_4_ plants initially fix atmospheric carbon in the mesophyll cells using phospho*enol*pyruvate carboxylase, an enzyme with a high effective affinity for CO_2_ (HCO_3_^−^ being the true substrate of the enzyme). Further four-carbon compounds (malate or aspartate) produced by this fixation are transported to the specialized bundle-sheath cells, where CO_2_ is released and fixed by Rubisco. Rubisco from C_4_ plants, which experiences ∼10-fold higher CO_2_ concentrations in bundle-sheath cells than does the enzyme in C_3_ plants [11], has a lower affinity for CO_2_ but a higher *k*_cat_ (≈4 s^−1^). Having less specific but faster Rubisco and no photorespiration losses, C_4_ plants require 60 to 75% less Rubisco to match the photosynthetic capacity of C_3_ plants [12], [13]. In fact, many C_4_ plants such as maize, sugarcane and sorghum are among the most productive of all species cultivated agriculturally. Although C_4_ plants appeared relatively recently in evolutionary terms and constitute only 3% of terrestrial plant species, they are already among the most successful and abundant groups in warm climates and are responsible for about 20% of terrestrial gross primary productivity [14], [15].

C_4_ photosynthesis evolved independently in at least 62 recognizable lineages of angiosperms and represents one of the most striking examples of a convergent biochemical adaptation in plants [16]. However, since its discovery, most attention has been devoted to the more numerous and agriculturally important C_4_ monocots in the Poaceae, while C_4_ eudicots have been studied less intensively. The family Amaranthaceae *sensu lato* (i.e. including Chenopodiaceae) [17], [18] contains about 180 genera and 2500 species, of which approximately 750 are C_4_ species [16], making it by far the largest C_4_ family among eudicots and the third-largest among angiosperms (after Poaceae and Cyperaceae). C_4_ photosynthesis evolved at least 15 times within Amaranthaceae [16] making this family a good model to study coevolution of C_4_ photosynthesis and Rubisco. Notably, the Amaranthaceae exceed the Poaceae and Cyperaceae in the diversity of photosynthetic organ anatomy [19], and is the only angiosperm family containing terrestrial C_4_ plants that lack Kranz anatomy, with three species having a single-cell rather than the more usual dual-cell C_4_ system [20], [21]. The predominantly tropical Amaranthaceae *sensu stricto* and primarily temperate and subtropical Chenopodiaceae have long been treated as two closely related families (see review in [19]) until the formal proposal that Chenopodiaceae should be included within the expanded Amaranthaceae based on a lack of separation between the two families in sequence data [17]. Amaranthaceae *sensu lato* (henceforth referred to as Amaranthaceae) constitutes the most diverse lineage of the Caryophyllales. Both C_3_ and C_4_ species from this family are adapted to a range of conditions from temperate meadows to the tropics, hot deserts and salt marshes. However, it has been shown that the abundance of C_4_ Amaranthaceae is correlated with precipitation but not temperature, in contrast to the abundance of C_4_ Poaceae and Cyperaceae, which is correlated with temperature but not precipitation [22].

Despite C_4_ Amaranthaceae showing different suites of anatomical and biochemical adaptations as well as ecological preferences compared to C_4_ Poaceae and Cyperaceae, like C_4_ monocots they possess faster but less CO_2_-specific Rubiscos than their C_3_ relatives [3], [5], [23]. Thus, Rubisco of C_4_ eudicots and monocots represents a notable example of convergent evolution of enzyme properties in phylogenetically distant groups. However, it is not known whether this functional convergence in Rubisco kinetics evolved via similar or different structural changes in protein [24]. Molecular adaptation can be inferred from comparison of the rates of non-synonymous (changing amino-acid protein sequence, *d*_N_) and synonymous (resulting in no change at the protein level, *d*_S_) mutations along a phylogenetic tree using maximum likelihood and Bayesian frameworks [25]. Recently, such methodology has been applied to the chloroplast gene *rbcL*, which encodes the large subunit of Rubisco that forms the enzyme's active site, and showed that positive Darwinian selection is acting within most lineages of plants [6]. Only a small fraction of Rubisco residues appear to be under positive selection, while most residues have been under purifying selection [6]. Some of these residues have been shown to be under positive selection within C_4_ lineages of Poaceae and Cyperaceae [26] and in the small Asteraceae genus, *Flaveria* [27], which contains both C_3_ and C_4_ species. However, no specific analysis has yet been made of Rubisco sequence evolution in a large group of C_4_ eudicots. In this study, we investigate positive selection on the *rbcL* gene of plants from the Amaranthaceae family and, in particular, focus on coevolution of Rubisco and C_4_ photosynthesis asking whether positive selection on the *rbcL* gene occured on branches leading to C_4_ clades and/or within C_4_ clades. Finally, we address the following question: which amino-acid replacements were associated with transitions from C_3_ to C_4_ photosynthesis in Amaranthaceae, and are these replacements unique to this lineage or shared with C_4_ monocots and/or *Flaveria*?

## Materials and Methods

### Phylogenetic analysis

**Figure 1:**
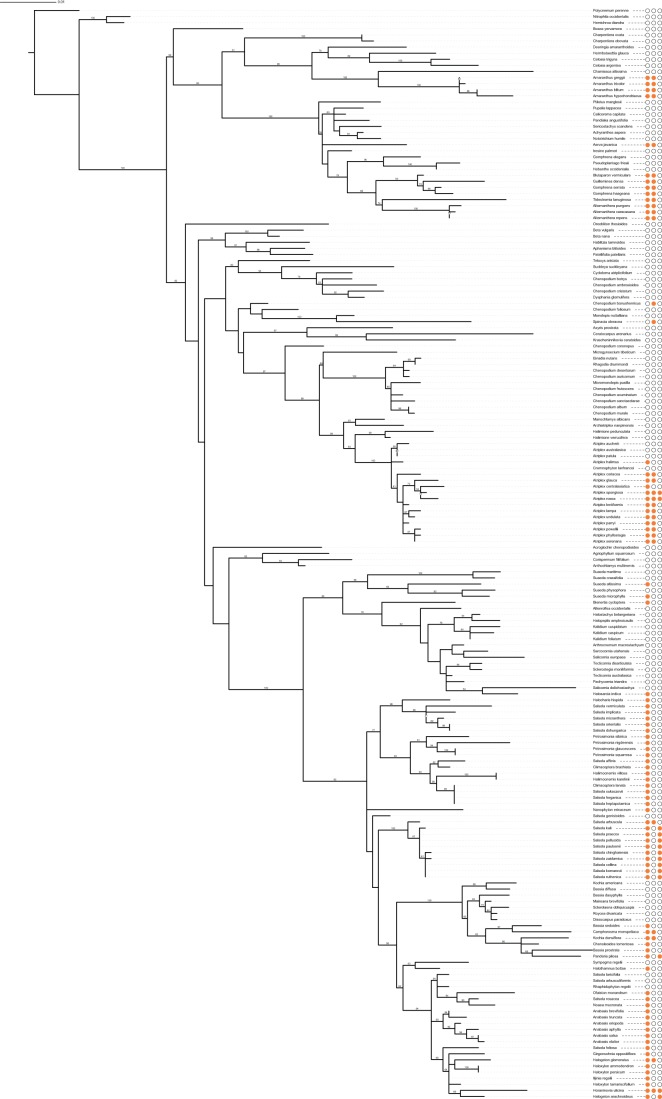
Maximum likelihood phylogram based on *rbcL* sequences of 179 Amaranthaceae species. Numbers above the branches are ML bootstrap support percentages. Filled orange circles of the first, second and third columns after species names indicate presence of C_4_ photosynthesis, serine at the position 281 and isoleucine at the position 309, respectively. The figure was composed using iTOL program [62].

We obtained all Amaranthaceae *rbcL* nucleotide sequences available in GenBank and aligned them. Sequences shorter than 1341 base pairs and sequences with missing data were excluded. The resulting trimmed alignment consisted of 179 *rbcL* sequences of 1341 base pairs long which represented 94% of the *rbcL* coding region and corresponded to positions 64 to 1404 of the *rbcL* sequence of *Spinacia oleracea* (GenBank AJ400848). The analysed dataset consisted of 95 C_3_ and 84 C_4_ species (Table S1). Most of the included sequences came from four studies [19], [28], [29], [30] and evenly represented all main lineages within the family (Fig. 1). Phylogeny was reconstructed using a maximum-likelihood inference (ML) conducted with RAxML version 7.2.6 [31] using the raxmlGUI interface [32]. We conducted five independent runs from different starting points to assess convergence within two likelihood units of the best tree, which was consistently selected. The parameters of partition were allowed to vary independently under the GTRGAMMA model of evolution as implemented in RAxML. ML nodal support was calculated by analysing 1000 bootstrap replicates. The best-scoring ML tree was used for tests of positive selection (see below).

### Tests for positive selection

Positive, neutral, or purifying selection at the molecular level can be inferred by comparing rates of non-synonymous (*d*_N_) and synonymous (*d*_S_) mutations along a phylogenetic tree [33]. Under neutrality, the two rates are expected to be equal (*d*_N_/*d*_S_ = 1), while purifying (negative) or adaptive (positive) selection is expected to deflate (*d*_N_/*d*_S_<1) or inflate (*d*_N_/*d*_S_>1) this ratio, respectively. One can use likelihood ratio tests to detect positive selection that affects only a subset of codons in a protein-coding gene, with positive selection indicated by accelerated nonsynonymous substitutions. Models assuming positive selection along all phylogeny or prespecified branches only (e.g. C_4_ lineages in our case) can be employed within Phylogenetic Analysis by Maximum Likelihood (PAML) framework [33].

We used the *codeml* program in the PAML v.4.4 package [33] to estimate *d*_N_/*d*_S_ ratio in the model M0, that allows for a single *d*_N_/*d*_S_ value across the whole phylogenetic tree obtained previously (see *Phylogenetic analyses* section). Further, *codeml* was used to perform likelihood ratio tests (LRTs) for positive selection among amino acid sites. The tree length value obtained from the model M0 was compared with tree length values obtained from other models to control for consistency among models. We performed two LRTs to compare null models which assume the same selective pressure along all branches of a phylogeny and do not allow positive selection (*d*_N_/*d*_S_ >1) with nested models which do allow it [33]. The first LRT, M1a-M2a, compares the M1a model (Nearly Neutral) which allows 0≤ *d*_N_/*d*_S_ ≤1 with the M2a model (Selection model; same as the M1a model plus an extra class under positive selection with *d*_N_/*d*_S_ >1). The second LRT, M8a-M8, compares the M8a model which assumes a discrete beta distribution for *d*_N_/*d*_S_, which is constrained between 0 and 1 including a class with *d*_N_/*d*_S_  = 1 with the M8 model which allows the same distribution as M8a but an extra class under positive selection with *d*_N_/*d*_S_ >1.

Finally, we performed two branch-site tests of positive selection along prespecified foreground branches [33], [34], [35]. The first was the A model for basal C_4_ branches only where positive selection was allowed only on branches leading to C_4_ clades. The second was the A model for all C_4_ branches where positive selection was allowed on branches leading to C_4_ clades and branches within C_4_ clades. The A1-A LRT compares the null model A1 with the nested model A. Both the A1 and A models allow *d*_N_/*d*_S_ ratios to vary among sites and among lineages. The A1 model allows 0< *d*_N_/*d*_S_ <1 and *d*_N_/*d*_S_  = 1 for all branches, and also two additional classes of codons with fixed *d*_N_/*d*_S_  = 1 along prespecified foreground branches while restricted as 0< *d*_N_/*d*_S_ <1 and *d*_N_/*d*_S_  = 1 on background branches. The alternative A model allows 0< *d*_N_/*d*_S_ <1 and *d*_N_/*d*_S_  = 1 for all branches, and also two additional classes of codons under positive selection with *d*_N_/*d*_S_ >1 along prespecified foreground branches while restricted as 0< *d*_N_/*d*_S_ <1 and *d*_N_/*d*_S_  = 1 on background branches. C_4_ lineages were marked as foreground branches.

For all LRTs, the first model is a simplified version of the second, with fewer parameters, and is thus expected to provide a poorer fit to the data (lower maximum likelihood). The M1a, M8a and A1 models are null models which do not allow codons with *d*_N_/*d*_S_ >1, whereas the M2a, M8 and A models are alternative models which do allow codons with *d*_N_/*d*_S_ >1. The significance of the LRTs was calculated assuming that twice the difference in the log of maximum likelihood between the two models was distributed as a chi-square distribution with the degrees of freedom (df) given by the difference in the numbers of parameters in the two nested models [34], [36]. For the M1a-M2a comparison df  = 2, and for M8a-M8, A1-A and M0 vs 2-rates model comparisons df  = 1. Each LRT was run two times using different initial *d*_N_/*d*_S_ values (0.1 and 0.4) to test for suboptimal local peaks. To identify amino acid sites potentially under positive selection, the parameter estimates from M2a, M8 and A models were used to calculate the posterior probabilities that an amino acid belongs to a class with *d*_N_/*d*_S_ >1 using the Bayes Empirical Bayes (BEB) approaches implemented in PAML [37]. Independently from *codeml* we used the SLR program which implements “sitewise likelihood-ratio” (SLR) method for detecting non-neutral evolution, a statistical test that can identify sites under positive selection even when the strength of selection is low [38]. The SLR test [38] consists of performing a likelihood-ratio test on a sitewise basis, testing the null model (neutrality, *d*_N_/*d*_S_  = 1) against an alternative model (*d*_N_/*d*_S_ ≠1). SLR method is a test of whether a given site has undergone selection or not, and the test statistic summarizes the strength of the evidence for selection rather than the strength of the selection itself [38]. The same input files with sequence alignment and species phylogeny were used for both *codeml* and SLR.

### Analysis of correlated evolution on phylogenies

Closely related taxa are not independent data points and they consequently violate the assumptions of conventional statistical methods [39]. Thus, we used analysis of correlated evolution on phylogenies to test the significance of correlation between pairs of discrete characters: (1) the presence/absence of C_4_ photosynthesis and (2) the presence/absence of particular amino-acid at sites found to be under positive selection along C_4_ branches in the A model of *codeml.* For this purpose, we used the phylogeny obtained using RAxML (see above) and performed Pagel's test of correlated (discrete) character evolution [40] implemented in the Mesquite package (version 2.72) [41]. Test was performed separately for each Rubisco residue under positive selection along C_4_ branches and Bonferroni correction was performed for simultaneous statistical testing.

### Structural analysis of Rubisco

We used the published Rubisco protein structure from spinach (*Spinacia oleracea*, Amaranthaceae) from data file 1RBO [42] obtained from the RCSB Protein Data Bank. Throughout the paper, the numbering of Rubisco large subunit residues is based on the spinach sequence. The locations and properties of individual amino acids in the Rubisco structure were analysed using DeepView – Swiss-PdbViewer v.3.7 [43] and by CUPSAT [44].

## Results

### Phylogenetic analysis

The ML phylogenetic tree (Fig. 1) for *rbcL* sequences from 179 Amaranthaceae species was largely congruent with previously obtained phylogenies and accepted taxonomic subdivisions of the family [19], [28], [29], [30], [45], [46], [47], [48]; however no statistical tests for topological similarity between our tree and previously published trees were performed because of different sizes and species compositions of datasets. A minimum of 16 independent origins of C_4_ photosynthesis were represented in the Amaranthaceae phylogeny if conservative approach for observed polytomies had been taken (Fig. 1), which is consistent with the estimate by Sage et al. [16]. The other assumption of this estimate was that no reversals from C_4_ to C_3_ were allowed. Predominance of C_4_ gains over reversals to C_3_ is supported by both empirical data and theoretical work [49].

### Tests for positive selection

Likelihood ratio tests (LRTs) for variation in *d*_N_/*d*_S_ ratios and for positive selection [33] were applied to the dataset of *rbcL* sequences from 179 C_3_ and C_4_ Amaranthaceae species. LRTs that were run using two different initial *d*_N_/*d*_S_ values (0.1 and 0.4) to test for suboptimal local peaks produced identical results. LRTs for positive selection [33] showed that the models assuming positive selection (M2a and M8) fit the data better than the nested models without positive selection (M1a and M8a; *p*-value <0.00001; Table 1). To test whether selection occurs specifically in C_4_ clades we used two branch site models (aka model A [33], [34]), one of which allowed positive selection only on branches leading to C_4_ clades and the other also allowed positive selection within the C_4_ clades. Each of these models was compared to an alternative model that allowed for no positive selection and only the latter of the two models demonstrated better fit to data than the model without positive selection (*p*-value <0.05; Table 1).

**Table 1:** Analysis of the Amaranthaceae *rbcL* genes for positively selected sites.

Model with positive selection a				Null model a			LRT d	
	log-likelihood	Parameters b	Positively selected sites c		log-likelihood	Parameters b	2*l*	*P*-value
**Analysis for positively selected sites common for C_3_ and C_4_ clades**								
M2a	−10711.44	*κ* = 3.00, *p*_0_ = 0.93, *ω*_0_ = 0.02, *p*_s_ = 0.01, *ω*_s_ = 2.62	*32*, 145, *279*, *439*	M1a	−10729.19	*κ* = 2.94, *p*_0_ = 0.93, *ω*_0_ = 0.02	35.5	<0.00001
M8	−10705.58	*κ* = 2.94, *p*_0_ = 0.96, *p* = 0.15, *q* = 3.04, *ω*_s_ = 1.56	*32*, 43, *145*, *225*, *262*, *279*, *439*, 443	M8a	−10717.70	*κ* = 2.90, *p*_0_ = 0.94, *p* = 0.20, *q* = 5.42	24.2	<0.00001
SLR	NA	*κ* = 2.75, *ω* = 0.10	*32*, 145, 225, *279*, *439*	NA	NA	NA	NA	NA
**Analysis for positively selected sites specific for branches leading to C_4_ clades**								
A	−10729.13	*κ* = 2.94, *p*_0_ = 0.93, *ω*_0_ = 0.02, *p*_s_ = 0.00, *ω*_s_ = NA	*no*	A1	−10729.13	*κ* = 2.94, *p*_0_ = 0.93, *ω*_0_ = 0.02	0.0	1.00000
**Analysis for positively selected sites specific for C_4_ clades**								
A	−10723.60	*κ* = 2.94, *p*_0_ = 0.92, *ω*_0_ = 0.02, *p*_s_ = 0.01, *ω*_s_ = 3.15	*281, 309*	A1	−10726.15	*κ* = 2.94, *p*_0_ = 0.92, *ω*_0_ = 0.02	5.1	0.02384

a M1a (nearly neutral), M2a (positive selection), M8a (beta & *ω = *1) and M8 (beta & *ω*) are PAML site models; A1 and A are PAML branch site models; SLR is “sitewise likelihood-ratio” method.

b *κ* is transition/transversion rate ratio; ω is *d*_N_/*d*_S_ ratio; *ω*_s_ is *d*_N_/*d*_S_ ratio in a class under putative positive selection; *p*_0_ and *p*_s_ are proportion of codons with *ω*<1 and *ω*>1, respectively; *p* and *q* are parameters of beta distribution in the range (0, 1); for the SLR test, the parameter values given are those optimal under M0.

c The sites listed are those at which positive selection is detected with a cutoff (significance level or posterior probability, as appropriate to the method used) >95%; those >99% are in italics. For the SLR test, the italic underlined sites are those at which there is still evidence for positive selection after correcting for multiple comparisons.

d LRT is likelihood ratio test, 2*l* is twice the difference of model log-likelihoods.

### Sites under positive selection

Four sites were identified as evolving under positive selection with a posterior probability >0.95 by BEB [37] implemented in the M2a model (residues 32, 145, 279, 439), but eight sites when BEB was implemented in the M8 model (all the same that in M2a plus sites 43, 225, 262, 443). Independent SLR analysis showed five sites evolving under positive selection (32, 145, 225, 279, 439), but only for one of them (site 279) evidence for positive selection remained significant after correcting for multiple comparisons. Two sites (residues 281 and 309) were shown to be under positive selection within C_4_ clades while under relaxed or purifying selection within C_3_ clades with a posterior probability >0.99 by BEB in the A model for C_4_ branches. Both sites had only two alternative amino acids in this dataset (Table 2). One of the two alternative amino acids was more frequent among C_4_ species, while the other was more frequent among C_3_ species (Table 2), but there were no fixed differences between C_4_ and C_3_ species. We refer to amino acids more frequently associated with C_4_ taxa as the ‘C_4_’ amino acids, but only for the sake of brevity, as they are not invariantly associated with C_4_ photosynthesis. Pagel's test of correlated character evolution [40] on phylogeny showed significant positive associations (*p*-value <0.05) between the presence of C_4_ photosynthesis and the presence of ‘C_4_’ amino acids at sites 281 and 309, shown to be under positive selection along C_4_ branches.

**Table 2:** Characteristics of amino-acid replacements under positive selection in the C_4_ lineages of Amaranthaceae.

AA No.^a^	AA changes ‘C_3_’→‘C_4_’			Type of changes b	ΔH^c^	ΔP^d^	ΔV^e^	SA^f^ (%)	ΔG^g^ (kJ/mol)	RFPS (%) h	% C_3_/% C_4_ species i	Location of residue	Structural motifs within 5 Å	Inter-actions j
														
281	A	**→**	S	HN **→** UP	−2.6	1.1	0.4	0.00	DS (−10.6)	2.7	2.1/34.5	Helix 4	Helices 4, 5	DD
309	M	**→**	I	HN **→** HN	2.6	−0.5	3.8	8.50	S (−1.3)	19.6	0.0/16.7	Strand F	Strand E; Helices F, 5	ID

a Amino acid (AA) numbering is based on the spinach sequence after [63].

b Side chain type changes. Types abbreviations: H – hydrophobic; N – nonpolar aliphatic; P – polar uncharged; U – hydrophilic (after [64]).

c Hydropathicity difference [65].

d Polarity difference [66].

e van der Waals volume difference [67].

f Solvent accessibility calculated using the spinach structure (pdb file 1RBO) by CUPSAT [44].

g Overall stability of the protein predicted using the spinach structure (pdb file 1RBO) by CUPSAT [44]. DS – destabilizing, S – stabilizing.

h RFPS – relative frequency of the particular residue to be under positive selection in C_3_ plants. Data from 112 *rbcL* datasets with detected positive selection from [6].

i Percentage of C_3_ and C_4_ species that have ‘C_4_’ amino acid among the 95 C_3_ species and 84 C_4_ species of Amaranthaceae analysed.

j Interactions in which the selected residues and/or residues within 5 Å of them are involved. ID – intradimer interactions; DD – dimer-dimer interactions (after [63]).

## Discussion

### Widespread positive selection on Rubisco

As the performance of Rubisco can directly affect plant growth and crop yields, substantial efforts have been made to study its structure and function, with the ultimate aim of trying to improve Rubisco performance [50]. The last few years have brought new approaches to improving our understanding of Rubisco evolution and its genetic mechanisms. The initial molecular-phylogenetic analysis of *rbcL* showed that positive selection is widespread among all main lineages of land plants, but is restricted to a relatively small number of Rubisco amino acid residues within functionally important sites [6]. Following studies showed that *rbcL* is under positive selection in particular taxonomic groups [26], [27], [51], [52], [53], [54], [55], [56]. Coevolution of residues is common in Rubisco of land plants as well as positive selection and there is an overlap between coevolving and positively selected residues [57]. Hence, phylogeny-based genetic analyses suggest there has been a constant fine-tuning of Rubisco to optimize its performance in specific conditions, in agreement with empirical observations that Rubisco enzymes from different organisms show diversity of kinetics better related to species ecology than phylogeny [4].

All eight residues shown under selection in Amaranthaceae using SLR and PAML models M2 and M8 were already shown to be under Darwinian selection in other groups of plants [6]. Five of these residues (145, 225, 262, 279 and 439) were among twenty most commonly selected Rubisco large subunit residues [6]. Findings in Amaranthaceae are in agreement with the previously described uneven distribution of putative fine-tuning residues in Rubisco [6]. Residues 43, 145, 225, 262 and 279 had only two alternative amino acids in the analyzed dataset, while residues 32 and 439 had three and residue 443 had four alternative amino acids. Residue 145 is involved in dimer-dimer interactions, residue 225 is involved in interactions with small subunit, while residue 262 is involved in both [8]. C_4_ photosynthesis has increased the availability of CO_2_ for Rubisco in numerous independently evolved lineages of C_4_ plants, including Amaranthaceae, driving selection for less specific but faster enzymes which have both higher *K*_M_(CO_2_) and *k*_cat_ values [3], [5], [23]. In the present study, we found that model A assuming positive selection on C_4_ branches provided a significantly better fit to the analysed Amaranthaceae dataset than the null model without selection (Table 1). We found no positive selection on branches which lead to C_4_ clades of Amaranthaceae, but we found positive selection specific for all C_4_ branches including branches which lead to C_4_ clades and branches within C_4_ clades (Table 1). This may be an argument in support of the hypothesis that C_3_ ancestors of C_4_ species, C_3_–C_4_ intermediates and C_4_ species at the dawn of their origin have Rubisco with C_3_ kinetics, but once C_4_ pump is fully functional it creates a strong selective pressure for acquiring Rubisco with C_4_ kinetics which then evolves during the stage of optimisation of C_4_ photosynthesis [58].

### Parallel amino-acid replacements in Rubisco from phylogenetically distant lineages

Bayesian analyses of *rbcL* sequences in a phylogenetic framework allowed us to identify two residues under directional selection along C_4_ branches within Amaranthaceae (Table 2). There are no common trends in physicochemical properties of ‘C_4_’ amino acids with respect to properties such as residue hydrophobicity, solvent accessibility, or location within the tertiary structure of the enzyme (Table 2). Alanine at the position 281 was replaced by serine at least eleven times within the studied species with nine of replacements taking place within C_4_ clades and two replacements in C_3_ species *Chenopodium bonus-henricus* and *Spinacia oleracea* (Fig. 1). Methionine at the position 309 was replaced by isoleucine at least four times, all of which within C_4_ clades (Fig. 1). Only three C_4_ species, *Atriplex spongiosa*, *A. rosea* and *Horaninovia ulicina*, had both ‘C_4_’ amino acids simulteniously. Seven C_4_ clades of which one was monospecific had ‘C_4_’ amino acids, while nine C_4_ clades of which six consisted of only one species did not have ‘C_4_’ amino acids (Fig. 1). More frequent occurrence of ‘C_4_’ amino acids in clades consisting of many species compared to monospecific clades corresponds to our findings of stronger positive selection within C_4_ clades (Table 1).

Interestingly, both selected residues in C_4_ Amaranthaceae are among the eight residues selected in C_4_ Cyperaceae and Poaceae [26] and the ‘C_4_’ amino acid 309I is also among selected in C_4_ *Flaveria* [27]. None of the ‘C_4_’ amino acids is fixed among C_4_ species, but they are more frequent among C_4_ lineages, ranging from 17 to 35% in C_4_ Amaranthaceae, and from 14 to 87% in C_4_ Cyperaceae and Poaceae (Table 2; percentage for C_4_ Cyperaceae and Poaceae calculated using numbers from [26]). Although ‘C_4_’ amino acids are not fixed among all C_4_ species, there is a significant positive association between their presence and C_4_ photosynthetic type in Amaranthaceae. Given the existence of C_4_ species without ‘C_4_’ amino acids , it is likely that other as yet unidentified amino acids replacements may be involved in Rubisco adaptation. The model of sequence evolution used to identify Rubisco residues under positive selection within C_4_ lineages averages selective pressure among selected branches (C_4_ branches in our case) and hence allows detection only of the most typical substitutions, potentially missing ones that are unique for a particular branch. Other possible explanations are variation in Rubisco kinetic properties not only between C_3_ and C_4_ groups of species but also within these groups [3], [4], [5], [23] and putative differences in other proteins which form the Rubisco complex (small subunit, Rubisco activase). Although the large subunits contain active sites, changes in small subunits may make significant contribution to kinetic properties of plant and algal Rubiscos [59], including differences observed between C_3_ and C_4_ plants [60], and the *rbcS* genes encoding small subunits have been shown under positive selection in C_4_ *Flaveria* [27].

Identical amino-acids in Rubisco of C_4_ Amaranthaceae and C_4_ Cyperaceae and Poaceae, representing eudicots and monocots with significantly different anatomy and ecological preferences [22], constitute a remarkable example of parallel molecular evolution in phylogenetically distant groups. This example becomes even more interesting if C_3_ plants are considered as well. Various groups of C_3_ plants such as some aquatic species and C_3_ species from cold habitats have faster but less CO_2_-specific Rubisco compared with their C_3_ relatives from terrestrial and warm conditions, respectively [3], [23]. Hence, some groups of C_3_ plants can arrive at the same evolutionary solutions for Rubisco fine-tuning as C_4_ plants. Indeed, ‘C_4_’ amino acids shown for C_4_ Amaranthaceae in the present study and for C_4_ monocots and *Flaveria* previously [26], [27], have been reported to be under positive selection in various groups of C_3_ plants by Kapralov and Filatov [6]. Moreover, residue 309 is among the most frequently positively selected sites in land plants, and although residue 281 itself is not, its close neighbours, residues 279 and 282, are among the most often positively selected ones [6]. Thus, we can conclude that both ‘C_4_’ amino acids, 281S and 309I, evolved in parallel in various phylogenetically distant lineages of C_3_ and C_4_ plants in which faster but less specific Rubisco was needed.

The residue 309 is located on the interface of large subunits within a large subunit dimer, while the residue 281 is involved into dimer-dimer interactions (Table 2). Methionine at position 309 is replaced by the smaller and more hydrophobic isoleucine, which has a stabilising and favourable effect on overall molecule stability according to CUPSAT calculations using spinach pdb-structure [44], while A281S replacement decreases hydrophobicy and may be destabilising (Table 2).

Effects of A281S replacement on kinetics of land plants Rubisco has not been studied, while recent study by Whitney et al. [61] using mutagenic approach showed that M309I replacement in *Flaveria* changed Rubisco kinetics from “C_3_-like” to “C_4_-like” making the enzyme faster but less CO_2_-specific. Importance of M309I replacement for changes in kinetics of *Flaveria* Rubisco was predicted using *in silico* approach similar to one used in the present study [27] and confirmed *in planta* by the study of Whitney et al. [61] making it a good case in support of further application of phylogeny-based methods for detecting residues under positive selection in Rubisco and elsewhere.

### Towards the periodic table of functional amino-acid replacements in Rubisco

Continuing population growth creating increasing demand for food, coupled with future climate change and its potentially dire consequences such as biome collapse and crop failure, both call for an improved understanding of mechanisms allowing plant species to adapt the photosynthetic process to a wide range of conditions. Hence, there is a necessity for more phylogeny-based studies of genes encoding Rubisco from various lineages of phototrophs established in different conditions to better understand Rubisco evolution at the molecular level. The integration of phylogenetic and biochemical research is required to study how Darwinian selection has created a range of enzymes with different kinetic and physical properties tailored to function in virtually all ecosystems on our planet. Knowledge of the role of specific residues in Rubisco adaptation to the particular conditions may provide clues for engineering better enzymes suited to contemporary agricultural needs as well as helping to understand what modifications in the enzyme may have been (and perhaps will be) driven by adaptation to different environmental conditions.

## Supporting Information

Table S1 **List of studied species.**
(XLSX)
